# High Unreported Mortality in Children and Youth (<25 Years) Living With HIV Who Were Lost to Care From Antiretroviral Therapy Programs in Southern Africa: Results From a Multicountry Tracing Study

**DOI:** 10.1097/QAI.0000000000003090

**Published:** 2022-09-09

**Authors:** Patience Nyakato, Benedikt Christ, Nanina Anderegg, Josephine Muhairwe, Laura Jefferys, Janneke van Dijk, Michael J. Vinikoor, Monique van Lettow, Cleophas Chimbetete, Sam J. Phiri, Matthias Egger, Marie Ballif, Constantin T. Yiannoutsos, Michael Schomaker, Reshma Kassanjee, Mary-Ann Davies, Morna Cornell

**Affiliations:** a Centre for Infectious Disease Epidemiology and Research, University of Cape Town, Cape Town, South Africa;; b Wellcome Centre for Infectious Diseases Research in Africa, Institute of Infectious Disease and Molecular Medicine;; c Institute of Social and Preventive Medicine, University of Bern, Bern, Switzerland;; d SolidarMed, Maseru, Lesotho;; e SolidarMed, Pemba, Mozambique;; f SolidarMed, Masvingo, Zimbabwe;; g Centre for Infectious Diseases Research in Zambia, Lusaka, Zambia;; h Dignitas International, Zomba, Malawi;; i Newlands Clinic, Harare, Zimbabwe;; j Lighthouse Trust Clinic, Lilongwe, Malawi;; k R.M. Fairbanks School of Public Health, Department of Biostatistics, Indiana University, Indianapolis, IN; and; l Department of Statistics, Ludwig-Maximilians-Universität München, München, Germany.

**Keywords:** HIV, mortality, loss to follow-up, misclassification, tracing

## Abstract

Antiretroviral therapy program mortality maybe underestimated if deceased patients are misclassified as lost.

**Methods:**

We used two-stage inverse probability weighting to account for probability of being: sampled for tracing and found by the tracer.

**Results:**

Among 680 children and youth aged <25 years on antiretroviral therapy who were lost and traced in Southern Africa between October 2017 and November 2019, estimated mortality was high at 9.1% (62/680). After adjusting for measured covariates and within-site clustering, mortality remained lower for young adults aged 20–24 years compared with infants aged <2 years [adjusted hazard ratio: 0.40 (95% confidence interval: 0.31 to 0.51)].

**Conclusions:**

Our study confirms high unreported mortality in children and youth who are lost and the need for tracing to assess vital status among those who are lost to accurately report on program mortality.

## Abstract 2

Supplemental Digital Content is Available in the Text.

## INTRODUCTION

Despite significant progress in pediatric HIV care and treatment, outcomes of children, adolescents, and young adults living with HIV (CAYHIV) aged <25 years continue to lag behind those in adults aged ≥25 years.^1,2^ CAYHIV have substantial loss to follow-up (LTFU) and mortality, especially in the first year after starting antiretroviral therapy (ART). LTFU hampers accurate outcome estimation as outcomes among those LTFU are unknown, which may bias the reporting of program performance.

Most studies reporting program outcomes estimate mortality at site-level from patient files, which are often incomplete or inaccurate. The International epidemiology Databases to Evaluate AIDS in Southern Africa collaboration (IeDEA-SA) has previously found that site-reported adult mortality can underestimate true mortality by up to 50%,^3^ with many undocumented deaths misclassified as LTFU. It is unclear whether the level of undocumented mortality among CAYHIV is similar, particularly given high rates of LTFU and the role of caregivers in retention of CAYHIV.^4^ In a large analysis of African and Asian pediatric programs, only 12/35 studies reported efforts to trace children LTFU, and none reported on outcomes among these children.^5^ We examined mortality from a large tracing study among CAYHIV considered LTFU in Southern Africa.^6^

## METHODS

### Setting and Sampling

We used data from a multicountry stratified sampling tracing study of adults and children among 7 ART programs in IeDEA-SA in rural and urban centers.^6–8^ For this analysis, we included CAYHIV aged ≤24 years initiating ART between 2004 and 2017 from 7 sites in 5 countries (Lesotho [1], Malawi [2], Mozambique [1], Zambia [1], and Zimbabwe [2]). The target sample number of adults and children was 500 participants per clinic, with predefined strata of age at last visit, sex, and time since ART initiation, but for clinics with fewer patients LTFU, the entire group was sampled (see Table S1, Supplemental Digital Content, http://links.lww.com/QAI/B959).^9^ Tracing was conducted using SMS, phone calls, and home visits between October 2017 and November 2019 (see Figure S1, Supplemental Digital Content, http://links.lww.com/QAI/B958).^6^ We defined children and youth as all children, adolescents, and young adults aged 0–24 years. We defined immunosuppression as per the WHO 2016 guidelines^10^ and defined ART as being on at least 3 antiretroviral therapy drugs.

### Outcomes

Our outcome of interest was all-cause mortality. We defined LTFU as having no recorded visit for ≥60 days (Malawi) and ≥ 90 days (all other sites) before start of tracing in keeping with local guidelines, and not known as deceased or transferred out. Death information was obtained through interviewing close informants including relatives or caregivers.

### Analysis

We summarized data using proportions, medians, interquartile ranges (IQRs), and rates.^11^ We used multivariate logistic regression to assess the factors associated with being found by the tracer. We applied weighted Weibull models to examine predictors of all-cause mortality with shared-frailty terms to account for the clustering within sites. Weights consisted of two-stage inverse probability weights to upweight those successfully traced accounting for the sampling strategy and likelihood of being found by the tracer to make results representative of all lost CAYHIV (methods to construct weights are explained elsewhere).^9,12,13^ We measured follow-up time from the last clinic visit to (1) death date if informed to have died, or (2) site-specific tracing date if found alive. We assumed all missing immune-suppression data (variable constructed from CD4 count/percent and age) were missing at random (MAR) and multiply-imputed these data with a chained equation approach.^14^ Results were combined using Rubin's^15^ rules. Analysis was performed in Stata version 17 (Stata Corporation, College Station, TX).

### Ethics Statement

The tracing study received ethical approval from the respective site/country institutional review boards (IRBs) for tracing and to transfer anonymized data to IeDEA-SA, which has approval from the University of Cape Town's Human Research Ethics Committee to receive and analyze these anonymized data.

## RESULTS

### Patient Characteristics

Of 972 CAYHIV recorded as lost and sampled for tracing, 121 (12.4%) were misclassified as lost, but were in care at the facility, 171 (17.6%) had missing files and both groups were therefore not included in the study. Among the remaining 680 CAYHIV traced, 3 in 5 were female, median age at last visit was 17.0 years (IQR: 5.4, 21.7), and nearly half (317, 46.6%) had been on ART for at least 12 months (Table 1). Health facilities were equally distributed between urban (344, 50.6%) and rural (336, 49.4%) areas and most were receiving ART at a health center (494, 67.3%) (see Table S1, Supplemental Digital Content, http://links.lww.com/QAI/B959).

**TABLE 1.** Patient Characteristics at Antiretroviral Therapy Start and Last Visit in Routine and Tracing Data

Variable	Traced (N = 680)	Vital status not confirmed (Remained LTFU) (N = 218)	Vital status confirmed (Alive/Dead) (N = 462)	*P* *
	n (%)	n (%)	n (%)	
Sex				0.560
Male	273 (40.2)	91 (41.7)	182 (39.4)	
Female	407 (59.9)	127 (58.3)	280 (60.6)	
Age at last visit, median (IQR) yrs	17.00 (5.40, 21.66)	15.68 (4.44, 21.61)	17.58 (6.31, 21.80)	0.306
0–<2	72 (10.6)	24 (11.0)	48 (10.4)	
2–<10	154 (22.7)	54 (24.8)	100 (21.7)	
10–<20	203 (29.9)	62 (28.4)	141 (30.5)	
≥20	251 (36.9)	78 (35.8)	173 (37.5)	
CD4 count at last visit, cells/µL	414 (253.5, 675)	464 (291, 610)	407.5 (233, 680)	0.516
0–199	54 (7.9)	11 (5.1)	43 (9.3)	
200–349	60 (8.8)	13 (6.0)	47 (10.2)	
350–499	63 (9.3)	16 (7.3)	47 (10.2)	
≥500	107 (15.7)	28 (12.8)	79 (17.1)	
Missing	396 (58.2)	150 (68.8)	246 (53.3)	
Year of ART start				0.074
2004–2013	127 (18.7)	31 (14.2)	96 (20.7)	
2014–2015	372 (54.7)	131 (60.1)	241 (52.2)	
2016–2017	181 (26.6)	56 (25.7)	125 (27.1)	
Duration on ART at last visit, mo				0.001
0–<6	263 (38.7)	114 (52.3)	141 (30.5)	
6–<12	100 (14.7)	41 (18.8)	63 (13.6)	
≥12	317 (46.6)	63 (28.9)	258 (55.8)	<0.0001
Country				
Lesotho	38 (5.2)	13 (6.0)	25 (5.4)	
Malawi	265 (36.1)	84 (38.5)	181 (39.2)	
Mozambique	131 (17.9)	22 (10.1)	109 (23.6)	
Zambia	136 (18.5)	87 (5.5)	49 (10.6)	
Zimbabwe	110 (15.0)	12 (5.5)	98 (21.2)	

* *P*-value between those traced and found and those who were not found.

### Tracing Outcomes

Overall, 246 (36.2%) remained LTFU, had missing contact details, or were not found in person (vital status was confirmed through an informant), 111 (16.3%) were alive and in care elsewhere (silent transfers), 141 (20.7%) had officially/silently transferred to other clinics, 120 (17.7%) were alive and out of care, and 62 (9.1%) had died (see Table S1, Supplemental Digital Content, http://links.lww.com/QAI/B959). Patient characteristics between those traced and found and those who were not found were similar except for CD4 count at last visit, duration on ART, and site of ART initiation (Table 1). Three in 5 participants found (confirmed vital status) and included in further analysis were female, median age at last visit was 17.6 years (IQR: 6.3, 21.8), most started treatment in 2014–2015, and over half had been on ART for at least 12 months. Longer duration on ART and initiating ART after 2013 were associated with higher odds of being successfully traced. The success of tracing also differed by country of ART initiation (see Figure S2, Supplemental Digital Content, http://links.lww.com/QAI/B958).

### Mortality Predictors

Overall, 462 participants had their vital status ascertained through the tracing and included in the analysis for mortality. Males had higher mortality than females [unadjusted rate: 13.3 (95% confidence interval (CI): 8.4 to 21.9) and 8.6 (95% CI: 4.4 to 19.3) per 100 py (Table 2). Mortality was highest for those who started ART at 0–<2 year old [16.5 (95% CI: 8.0 to 34.1)] per 100 py and declined with higher age groups [20–24 years: 3.2 (95% CI: 1.1 to 13.2) per 100 py]. Children and youth who were immune suppressed at last visit also had higher mortality rates compared with those who were not [21.8 (95% CI: 10.7 to 56.3) vs 8.9 (95% CI: 2.3 to 64.9)]. Mortality rates were lowest for those ≥12 months on ART.

**TABLE 2.** Predictors of Mortality Among Children and Youth Living With HIV Who Are Successfully Traced

Patient Characteristics	No. of Events	Crude rates (per 100 person years) (95% CI)	Crude HR (95% CI)	Adjusted HR* (95% CI)
Sex				
Male	33	13.34 (8.42 to 21.90)	Ref	Ref
Female	29	8.60 (4.40 to 19.28)	0.65 (0.25 to 1.69)	0.90 (0.62 to 1.30)
Age at last visit, yrs				
0–<2	17	16.52 (7.99 to 34.07)	Ref	Ref
2–<10	20	7.35 (4.12 to 13.99)	0.89 (0.63 to 1.26)	0.84 (0.65 to 1.08)
10–<20	14	3.02 (0.88 to 14.22)	0.51 (0.13 to 2.05)	0.27 (0.09 to 1.04)
20–24	11	3.16 (1.05 to 13.16)	0.36 (0.30 to 0.44)	**0.40 (0.31** to **0.51)**
Immunosuppression at last visit				
No	4	8.88 (2.25 to 64.87)	Ref	Ref
Yes	16	21.79 (10.68 to 56.25)	1.80 (0.68 to 4.74)	1.64 (0.66 to 4.05)
Duration on ART, mo				
0–<6	15	12.27 (6.50 to 25.67)	Ref	Ref
6–<12	12	44.75 (16.72 to 185.43)	2.48 (1.03 to 5.98)	2.70 (0.86 to 8.47)
≥12	35	7.45 (1.35 to 6.53)	0.12 (0.01 to 2.35)	0.13 (0.01 to 1.30)
Location of health facility				
Rural	40	7.74 (4.28 to 15.04)	Ref	Ref
Urban	22	13.47 (6.96 to 30.59)	2.18 (0.26 to 18.14)	2.24 (0.13 to 40.11)

* Adjusted for country where health facility is located.

HR, hazard ratio.

After adjusting for measured covariates and within-site clustering, mortality remained lowest for young adults aged 20–24 years compared with infants aged <2 years [adjusted Hazard ratio: 0.4 (95% CI: 0.3 to 0.5)].

## DISCUSSION

In our study, through tracing, the vital status of nearly 40% of all CAYHIV could not be ascertained because they could not be traced, nearly 20% were alive and out of care, 21% had silently transferred to other clinics, and about a 10th had died. The estimated mortality was high at 9.1% (62/680). We also found that longer duration on ART and initiating ART after 2013 were associated with higher odds of being successfully traced. The success of tracing also differed by country of ART initiation. Higher mortality rates were found among infants aged below 2 years compared with young adults aged 20–24 years.

To the best of our knowledge, this is the first multicountry tracing study among CAYHIV considered LTFU. The high rates of unreported mortality are consistent with results from a systematic review,^4^ and a multiregional tracing study,^16^ although mortality in our study was lower than a Zambian tracing study in which 26% of children had died.^17^ The children in the Zambian study were younger (0–15 years) compared with those in our study (0–24 years) and as reported, younger children have a higher risk of mortality compared with older children. Mortality estimates among adults who are LTFU and traced, vary across settings from 6% in Mozambique^18^ to 30% in Malawi^19^ with other studies reporting no differences.^20^ The high mortality among children and youth LTFU could be patients who were too ill to return to care and died immediately after being lost (ie, they are lost because they have actually died), or may have stopped taking ART after their missed appointment, leading to nonsuppression, disease progression, and mortality (ie, they died because they are lost and no longer on treatment).^12^ In our study, most of the children and youth died within 1 year from their last visit [median (IQR): 0.4 (0.2, 1.3) years] suggesting that they were disengaged from care despite being ill. There were 27/62 (44%) of deaths that occurred within the first 3 months from the last clinic visit, and since visits were scheduled three-monthly, these appear to have missed their visits because they were deceased. The most common risk factors associated with LTFU in the literature are psychosocial, structural, and clinical barriers. Psychosocial barriers include denial of HIV status and nondisclosure; structural barriers include economic factors such as lack of transport costs, long distance between clinic and patient's home and clinical factors may include drug stockouts, long waiting times at the clinic, and health care workers' attitudes.^17,19^

Mortality was highest among the youngest children. Infants are more vulnerable to severe disease progression and HIV-related complications. Without ART, only half survive to their second birthday.^21–23^ Despite the introduction of early infant diagnosis (EID) and earlier initiation of ART, many children are only diagnosed during hospital admission with advanced disease and a high risk of mortality.^24^ ART programs focused on EID and retention of these children should be strengthened to reduce mortality. Our study provides additional evidence that the first 6 months on ART are critical for survival.^25,26^ Longer duration on ART would also imply sustained viral suppression and hence reduced risk of mortality in this age group. ART programs should ensure that CAYHIV achieve and maintain high CD4 counts and stay on ART, and trace those who are lost as soon as they miss a clinic visit. ART programs should also prioritize children and youth who enter care with severe disease to ensure improved survival in the first 6 months on ART. Interventions to prevent LTFU among the youngest children and in the first 6 months after ART start are urgently needed.

Our study was strengthened by the large sample of traced CAYHIV from 5 countries in Southern Africa. Our results should be generalizable to pediatric HIV programs with high rates of LTFU and mortality in low- and middle-income countries. Our study was limited by missing CD4 count/percent measurements, which we addressed using multiple imputation. We did not have data on the causes of deaths and were also unable to link to national registers for any deaths that may have occurred among those that we were unable to find through tracing.

In conclusion, our study adds much-needed evidence on outcomes of CAYHIV who are LTFU, confirms high mortality in those lost, and the need for tracing to assess vital status among those who are lost to accurately report on program mortality. Treatment programs must prioritize retaining CAYHIV who start ART below 2 years old and those in the first year of initiating ART, and actively trace them should they miss a clinic visit.

## Supplementary Material

SUPPLEMENTARY MATERIAL

## References

[1] Davies M-A Pinto J. Targeting 90-90-90–don't leave children and adolescents behind. J Int AIDS Soc. 2015;18(7 suppl 6):20745. 26639121 10.7448/IAS.18.7.20745 PMC4670834

[2] HIV/AIDS JUNPo. Ending AIDS: Progress towards the 90-90-90 Targets. Global AIDS Update. Geneva, Switzerland: UNAIDS Secretariat; 2017.

[3] Boulle A Schomaker M May MT . Mortality in patients with HIV-1 infection starting antiretroviral therapy in South Africa, Europe, or North America: a collaborative analysis of prospective studies. PLoS Med. 2014;11:e1001718. 25203931 10.1371/journal.pmed.1001718 PMC4159124

[4] Fox MP Rosen S. Systematic review of retention of pediatric patients on HIV treatment in low and middle-income countries 2008–2013. AIDS. 2015;29:493–502. 25565496 10.1097/QAD.0000000000000559

[5] Abuogi LL Smith C McFarland EJ. Retention of HIV-infected children in the first 12 months of anti-retroviral therapy and predictors of attrition in resource limited settings: a systematic review. PLoS One. 2016;11:e0156506. 27280404 10.1371/journal.pone.0156506 PMC4900559

[6] Ballif M Christ B Anderegg N . Tracing people living with HIV who are lost to follow-up at ART programs in Southern Africa: a sampling-based cohort study in six countries. Clin Infect Dis. 2022;74(2):171–179. 33993219 10.1093/cid/ciab428 PMC8800181

[7] Egger M Ekouevi DK Williams C . Cohort profile: the International epidemiological Databases to Evaluate AIDS (IeDEA) in sub-Saharan Africa. Int J Epidemiol. 2012;41:1256–1264. 21593078 10.1093/ije/dyr080 PMC3465765

[8] Chammartin F Ostinelli CHD Anastos K . Cohort profile: the International epidemiology Databases to Evaluate AIDS (IeDEA) in sub-Saharan Africa, 2012–2019. BMJ Open. 2012;10:e035246. 10.1136/bmjopen-2019-035246 PMC7232622 32414825

[9] Christ B Ballif M Anderegg N . Tracing people living with HIV who are lost to follow-up at ART programs in Southern Africa: Study protocol. Oxford, England: Clinical Infectious Diseases, Oxford University Press; 2020.

[10] World Health Organization. Consolidated Guidelines on the Use of Antiretroviral Drugs for Treating and Preventing HIV Infection: Recommendations for a Public Health Approach. Geneva, Switzerland: World Health Organization; 2016. 27466667

[11] Heumann C Schomaker M. Introduction to Statistics and Data Analysis. Cham, Switzerland: Springer Nature Switzerland AG; 2016.

[12] Schomaker M Gsponer T Estill J . Non‐ignorable loss to follow‐up: correcting mortality estimates based on additional outcome ascertainment. Stat Med. 2014;33:129–142. 23873614 10.1002/sim.5912 PMC3859810

[13] Nyakato P Kiragga AN Kambugu A . Correction of estimates of retention in care among a cohort of HIV-positive patients in Uganda in the period before starting ART: a sampling-based approach. BMJ open. 2018;8:e017487. 10.1136/bmjopen-2017-017487 PMC5914905 29678963

[14] White IR Royston P Wood AM. Multiple imputation using chained equations: issues and guidance for practice. Stat Med. 2011;30:377–399. 21225900 10.1002/sim.4067

[15] Rubin DB. Inference and missing data. Biometrika. 1976;63:581–592.

[16] Kassanjee R Johnson LF Zaniewski E ; International epidemiology Databases to Evaluate AIDS IeDEA Collaboration. Global HIV mortality trends among children on antiretroviral treatment corrected for under‐reported deaths: an updated analysis of the International epidemiology Databases to Evaluate AIDS collaboration. J Int AIDS Soc. 2021;24:e25780. 34546646 10.1002/jia2.25780 PMC8454681

[17] Mutanga JN Mutembo S Ezeamama AE . Predictors of loss to follow-up among children on long-term antiretroviral therapy in Zambia (2003–2015). BMC Public Health. 2019;19:1120. 31416432 10.1186/s12889-019-7374-0 PMC6694674

[18] Anderegg N Hector J Jefferys LF . Loss to follow-up correction increased mortality estimates in HIV–positive people on antiretroviral therapy in Mozambique. J Clin Epidemiol. 2020;128:83–92. 32828836 10.1016/j.jclinepi.2020.08.012 PMC7957226

[19] Tweya H Feldacker C Estill J . Are they really lost? “True” status and reasons for treatment discontinuation among HIV infected patients on antiretroviral therapy considered lost to follow up in urban Malawi. PLoS One. 2013;8:e75761. 24086627 10.1371/journal.pone.0075761 PMC3784425

[20] Zürcher K Mooser A Anderegg N ; IeDEA and MESH Consortia. Outcomes of HIV‐positive patients lost to follow‐up in African treatment programmes. Trop Med Int Health. 2017;22:375–387. 28102610 10.1111/tmi.12843 PMC5580236

[21] Newell M-L Coovadia H Cortina-Borja M ; Ghent International AIDS Society (IAS) Working Group on HIV Infection in Women and Children. Mortality of infected and uninfected infants born to HIV-infected mothers in Africa: a pooled analysis. Lancet. 2004;364:1236–1243. 15464184 10.1016/S0140-6736(04)17140-7

[22] Kranzer K Bradley J Musaazi J . Loss to follow-up among children and adolescents growing up with HIV infection: age really matters. J Int AIDS Soc. 2017;20:21737. 28715158 10.7448/IAS.20.1.21737 PMC5577636

[23] Violari A Cotton MF Gibb DM ; CHER Study Team. Early antiretroviral therapy and mortality among HIV-infected infants. N Engl J Med. 2008;359:2233–2244. 19020325 10.1056/NEJMoa0800971 PMC2950021

[24] Abrams EJ Woldesenbet S Soares Silva J . Despite access to antiretrovirals for prevention and treatment high rates of mortality persist among HIV-infected infants and young children. Pediatr Infect Dis J. 2017;36:595–601. 28027287 10.1097/INF.0000000000001507 PMC5432395

[25] Davies MA Gibb D Turkova A. Survival of HIV-1 vertically infected children. Curr Opin HIV AIDS. 2016;11:455–464. 27716730 10.1097/COH.0000000000000303 PMC5384722

[26] Leroy V Malateste K Rabie H . Outcomes of antiretroviral therapy in children in Asia and Africa: a comparative analysis of the IeDEA pediatric multiregional collaboration. J Acquir Immune Defic Syndr. 2013;62:208. 23187940 10.1097/QAI.0b013e31827b70bf PMC3556242

