# Supplementary material for: High Unreported Mortality in Children and Youth (<25 Years) Living With HIV Who Were Lost to Care From Antiretroviral Therapy Programs in Southern Africa: Results From a Multicountry Tracing Study
Source: J Acquir Immune Defic Syndr. 2022 Sep 9;91(5):429–33. doi: 10.1097/QAI.0000000000003090 (PMC9646412; doi:10.1097/QAI.0000000000003090)

**Supplementary Figure 1: Flow chart showing the tracing process**

No vital & care status obtained

No

Yes

Patient file not found

Check patient files:

Is “lost to follow-up” confirmed?

List of randomly sampled PLWH on ART that are LTFU

Vital & care status obtained

Up to three home visits

One text message

Vital & care status obtained

**No tracing done**

Tracing impossible

Tracing not needed

**Tracing done**

Vital status obtained but no care status obtained

No vital & care status obtained

Tracing not successful

Tracing partly successful

Tracing successful

Found in person

Were PLWH LTFU found in person, found through an informant or not found?

Found through informant

Not found

Up to three phone calls

One additional phone call/
home visit

Legend
 Tracing methods
 Outcomes not or not fully obtained
 Outcomes obtained

**Supplementary Figure 2: Multivariate logistic regression model showing factors associated with being found by the tracer on a log odds scale**


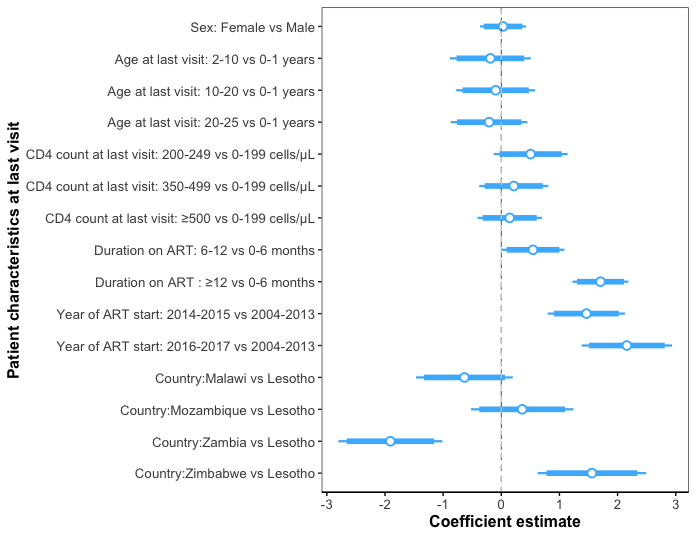

Supplement: SUPPLEMENTARY MATERIAL [file qai-91-429-s001.docx]
