# Supplementary material for: High Unreported Mortality in Children and Youth (<25 Years) Living With HIV Who Were Lost to Care From Antiretroviral Therapy Programs in Southern Africa: Results From a Multicountry Tracing Study
Source: J Acquir Immune Defic Syndr. 2022 Sep 9;91(5):429–33. doi: 10.1097/QAI.0000000000003090 (PMC9646412; doi:10.1097/QAI.0000000000003090)
Supplement: SUPPLEMENTARY MATERIAL [file qai-91-429-s002.docx]

**Supplementary Table 1: Description of tracing variables among patients that were traced**

|  | **Number**  **(N=680)** | **Percentage (%)** |
| --- | --- | --- |
| **Location of the health facility** |  |  |
| Rural | 336 | 49.4% |
| Urban | 344 | 50.6% |
| **Level of care at health facility** |  |  |
| Health centre | 494 | 67.3% |
| District hospital/health centre | 186 | 25.3% |
| Regional, provincial or university hospital | 54 | 7.4% |
| **Method of tracing used** |  |  |
| SMS (text messages) | 96 | 13.8% |
| Phone call | 139 | 27.7% |
| Home visit | 407 | 58.5% |
| **Ascertained tracing outcomes** |  |  |
| Dead | 62 | 9.1% |
| Retained in care | 111 | 16.3% |
| Transfer-out (self/ official) | 141 | 20.7% |
| Out of care | 120 | 17.7% |
| Unknown (not found in person/missing contact details/LTFU) | 246 | 36.2% |
| **Tracing outcome details among those known to be alive** |  |  |
| Patient never missed a clinic visit | 32 | 8.0% |
| Patient returned to care at the facility | 79 | 19.8% |
| Patient in care at another facility | 141 | 35.3% |
| Patient stopped taking ART | 117 | 29.3% |
| Patient never started ART | 3 | 0.8% |
| Patient/caregiver refused to be interviewed | 3 | 0.8% |
| Tracing attempted but patient not found in person | 25 | 6.3% |
| **Care giver relationship with CAHIV** |  |  |
| Parent | 72 | 27.5% |
| Grand parent | 46 | 17.6% |
| Spouse/ sexual partner | 20 | 7.6% |
| Sibling | 64 | 24.4% |
| Child | 9 | 3.4% |
| Other relative | 14 | 5.3% |
| Other | 28 | 10.7% |
| Missing | 9 | 3.4% |
| **Time not seen from last visit until study start, months** |  |  |
| 0-5 | 39 | 5.8% |
| 6-11 | 79 | 11.8% |
| 12-23 | 238 | 35.4% |
| 24-35 | 150 | 22.3% |
| ≥ 36 | 166 | 24.7% |
